# Impact of Selected Signaling Proteins on SNAIL 1 and SNAIL 2 Expression in Ovarian Cancer Cell Lines in Relation to Cells’ Cisplatin Resistance and EMT Markers Level

**DOI:** 10.3390/ijms22020980

**Published:** 2021-01-19

**Authors:** Michal Kielbik, Izabela Szulc-Kielbik, Magdalena Klink

**Affiliations:** Institute of Medical Biology, Polish Academy of Sciences, 106 Lodowa Str., 93-232 Lodz, Poland; iszulc@cbm.pan.pl (I.S.-K.); mklink@cbm.pan.pl (M.K.)

**Keywords:** ovarian cancer, SNAIL 1, SNAIL 2, cadherins, signaling proteins, chemoresistance

## Abstract

It has been increasingly recognized that SNAIL1 and SNAIL2, as major EMT-inducers, might also be involved in drug resistance of cancer cells. We sought to determine a relation between SNAIL1/2, E-cadherin and N-cadherin expression, as well as ovarian cancer cells’ resistance to cisplatin and EMT markers’ level. Thus, four ovarian cancer cell lines, were used: A2780, A2780cis, SK-OV-3 and OVCAR-3. We assessed the impact of ERK1/2, AKT and STAT3 proteins (chosen by the profiling activity of over 40 signaling proteins) on SNAIL1/2 expression, along with E-cadherin and N-cadherin levels. We showed that expression of SNAIL1 and N-cadherin are the highest in cisplatin-resistant A2780cis and SK-OV-3 cells, while high SNAIL2 and E-cadherin levels were observed in cisplatin-sensitive A2780 cells. The highest E-cadherin level was noticed in OVCAR-3 cells. SNAIL1/2 expression was dependent on ERK1/2 activity in cisplatin-resistant and potentially invasive SK-OV-3 and OVCAR-3 cells. STAT-3 regulates expression of SNAIL1/2 and leads to the so-called “cadherin switch” in cancer cells, independently of their chemoresistance. In conclusion, SNAIL1, but not SNAIL2, seems to be involved in ovarian cancer cells’ cisplatin resistance. STAT3 is a universal factor determining the expression of SNAIL1/2 in ovarian cancer cells regardless of their chemoresitance or invasive capabilities.

## 1. Introduction

The accepted standard of the first-line chemotherapy of epithelial ovarian cancer (EOC) covers the combination of a platinum-containing agent (cisplatin, carboplatin) and a member of taxanes (paclitaxel, docetaxel). Although the initial response to chemotherapy is promising, with the majority of patients (70%) responding positively, most of these patients experience recurrence of disease, with the incidence varying from up to 30% in early stage to up to 85% in advanced stage of disease [1,2]. Very often, the recurrent ovarian cancer has acquired resistance to chemotherapeutic agents. Moreover, it has been proven that the creation of metastases is common in ovarian cancer, as this disease exhibits high invasiveness and migration potential [3]. Metastatic sites differ from primary tumor in response to chemotherapeutic agents, as invading cancer cells are characterized by enhanced chemoresistance (either innate or acquired), which further limits the effectiveness of treatment [4].

Since the majority of malignant tumors are of epithelial origin, the ability to change their morphology and phenotype is required in order to disseminate from primary tumor and invade distant sites in organism. In this regard, the epithelial to mesenchymal transition (EMT) process is absolutely essential and a key part of the metastatic cascade event. It allows tumor cells to adopt a mesenchymal phenotype, thereby increasing their motility, as well as apoptotic resistance, and allows for their detachment and initial spread within the local extracellular matrix [5]. The EMT process is determined by a complex network of transcription factors, among which, those belonging to the SNAIL family (SNAIL 1, 2, 3), basic helix–loop–helix (bHLH) family (TWIST 1 and TWIST 2) and zinc-finger E-box-binding homeobox family (ZEB 1 and ZEB 2) seem to have the leading roles. They are often termed “master regulators” of EMT, since their activity leads to the downregulation of epithelial genes and upregulation of genes associated with mesenchymal phenotype [6,7]. Interestingly, EMT is also known to contribute to acquired chemoresistance in diverse types of cancers, including ovarian cancer [8,9,10,11]. Moreover, the manipulation of “master regulators” expression modulates the response of cancer cells to chemotherapeutic drugs [12,13]. On the other hand, chemotherapy, apart from inducing resistance to chemotherapeutics, can also cause cancer cells to undergo EMT. Thus, the EMT/chemoresistance axis is involved in the cancer disease progression and patients’ response to therapy [12,14]. However, the exact interplay between chemoresistance and EMT, especially in ovarian cancer, is not fully clear and requires more extensive study. Moreover, the exact molecular mechanisms responsible for the induction of both phenomena in ovarian cancer cells have not been clarified.

SNAIL 1 and SNAIL 2 are the most studied proteins in various types of cancer and it has been proven that their level correlates with the increased migration, invasion and metastasis of cancer cells [15,16,17,18]. The primary function of SNAIL 1 and SNAIL 2 is the induction of EMT by repressing E-cadherin transcription through interaction with its gene—*CDH1*. They can also repress other epithelial markers (occluding, claudin) or tumor suppressors (PTEN) and simultaneously upregulate mesenchymal markers (Vimentin, Fibronectin), as well as the activity of metalloproteinases [19,20]. In addition to being EMT inducers, SNAIL 1 and SNAIL 2 are also implicated in the ovarian cancer cells’ resistance to platinum compounds [13]. The expression of SNAIL transcription factors undergoes complex regulation on many levels, among which post-translational modifications of their central domain, resulting in either phosphorylation, oxidation or nuclear export, modulate their stability and cellular location. These modifications are a direct result of various signaling cascade activities, among which the most noteworthy are definitely those involving TGF-β/SMAD proteins, phosphoinositide 3-kinase (PI3K)/AKT kinase, mitogen-activated protein kinases (MAPKs), Wnt/GSK3-β and the signal transducer and activator of transcription 3 (STAT3) [19,21,22].

In this study, we sought to determine whether there are any significant differences in the expression of SNAIL 1 and SNAIL 2 between four commercially available ovarian cancer cell lines (A2780, A2780cis, SK-OV-3 and OVCAR-3), which differ in the degree of chemoresistance and invasive potential. Secondly, we aimed to assess which of the selected intracellular signaling pathways are the most impactful on the SNAIL 1 and SNAIL 2 expression and the level of EMT markers (E-cadherin and N-cadherin). Lastly, we wanted to clarify whether potential changes in SNAIL 1 and SNAIL 2 expression, caused by blocking chosen signaling proteins’ activity, affect the ovarian cancer cells’ response to cisplatin.

## 2. Results

### 2.1. EC_50_ Value of Cisplatin for Each Ovarian Cancer Cell Line

**Figure 1:**
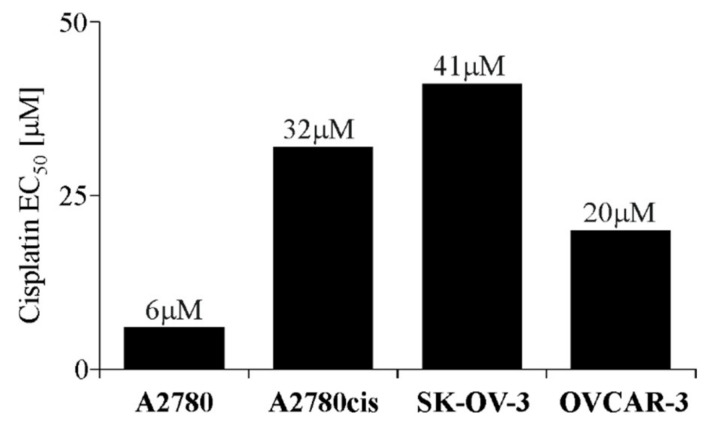
Effective concentration of cisplatin (EC_50_) for A2780, A2780cis, SK-OV-3 and OVCAR-3 cell lines. All cell lines were cultured with various concentrations of cisplatin (1–200 µM) or not (control) for 48 h. Cell viability was determined with MTT assay and is presented as the EC_50_ value for each cell line, calculated from three independent experiments (*n* = 3).

In the first stage of this research, we evaluated the cisplatin’s half maximal effective concentration (EC_50_) values for A2780, A2780cis, SK-OV-3 and OVCAR-3 cell lines after 48 h of exposure. The obtained results (Figure 1) indicate that SK-OV-3 and A2780cis cell lines are the most cisplatin resistant (EC_50_ = 41 and 32 µM, respectively), being almost two times more resistant than the OVCAR-3 cell line (EC_50_ = 20 µM) and up to six times more resistant than the most cisplatin-sensitive A2780 cells (EC_50_ = 6 µM).

### 2.2. Basal Expression of SNAIL 1 and SNAIL 2 in Ovarian Cancer Cell Lines

**Figure 2:**
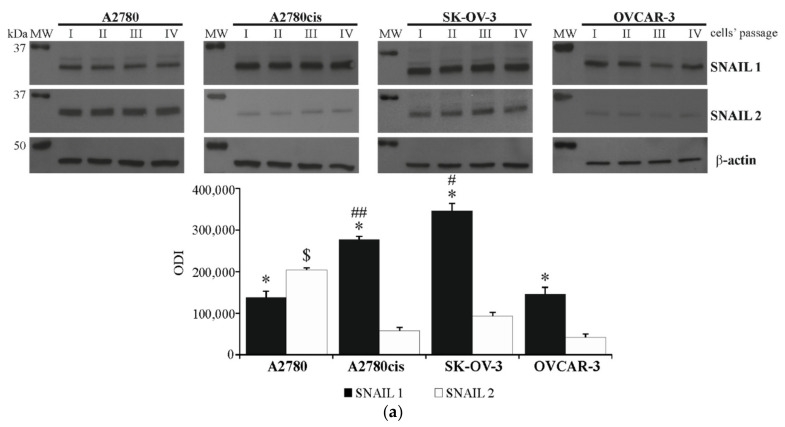

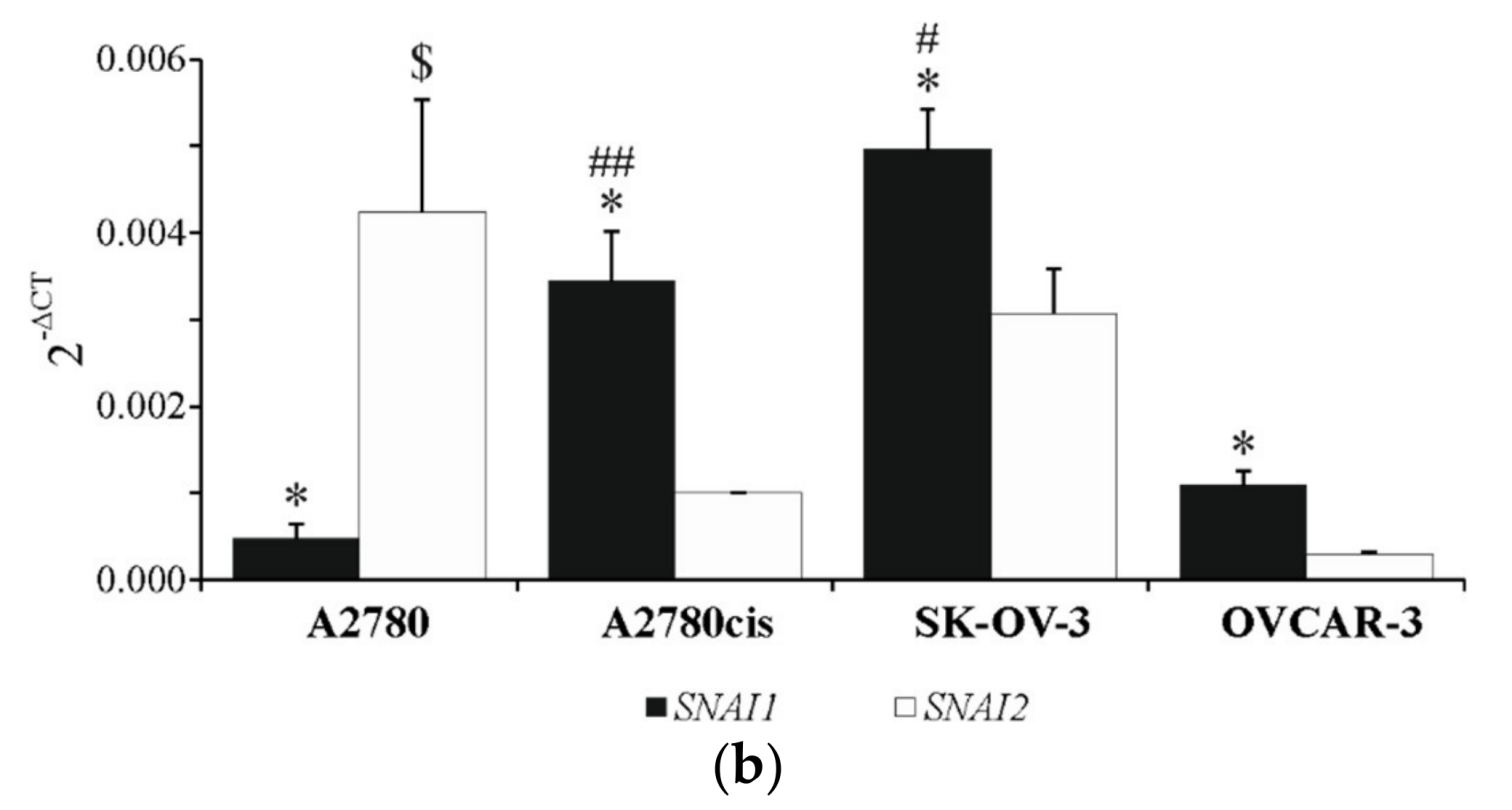
The expression of SNAIL 1 and SNAIL 2 in A2780, A2780cis, SK-OV-3 and OVCAR-3 cell lines. (**a**) The basal levels of SNAIL 1 and SNAIL 2 proteins were determined with immunoblotting-ECL. Representative immunoblots of SNAIL 1 and SNAIL 2, along with β-actin level, are presented. The acquired bands were quantified by densitometric analysis and data are presented as the mean optical density intensity (ODI) ± SD from four independent experiments (*n* = 4). * Statistically significant difference in SNAIL 1 and SNAIL 2 level: SNAIL 1 vs. SNAIL 2 in A2780, A2780, SK-OV-3 or OVCAR-3 cell line, *p* ≤ 0.03 (Mann–Whitney *U* test test). ## Statistically significant difference in SNAIL 1 level: A2780cis vs. A2780 or OVCAR-3, *p* ≤ 0.03 (Mann–Whitney *U* test) # Statistically significant difference in SNAIL 1 level: SK-OV-3 vs. A2780 or OVCAR-3, *p* ≤ 0.03 (Mann–Whitney *U* test). $ Statistically significant difference in SNAIL 2 level: A2780 vs. A2780cis or SK-OV-3 or OVCAR-3, *p* ≤ 0.03 (Mann–Whitney *U* test). (**b**) The basal expression of *SNAI1* and *SNAI2* genes was determined with real-time PCR assay. Data are presented as mean 2^−ΔCT^ ± SD from four independent experiments (*n* = 4). 2^−ΔCT^ represents an absolute value of target mRNA level, in particular, cell line. * Statistically significant difference in *SNAI1* and *SNAI2* level: *SNAI1* vs. *SNAI2* in A2780, A2780, SK-OV-3 or OVCAR-3 cell line, *p* ≤ 0.04 (Mann–Whitney *U* test). ## Statistically significant difference in *SNAI1* level: A2780cis vs. A2780 or OVCAR-3, *p* ≤ 0.03 (Mann–Whitney *U* test) # Statistically significant difference in *SNAI1* level: SK-OV-3 vs. A2780 or OVCAR-3, *p* ≤ 0.03 (Mann–Whitney *U* test). $ Statistically significant difference in *SNAI2* level: A2780 vs. A2780cis or SK-OV-3 or OVCAR-3, *p* ≤ 0.03 (Mann–Whitney *U* test).

In the second stage of this research, the basal level of SNAIL 1 and SNAIL 2 proteins, as well as the basal expression of SNAI1 and SNAI2 genes were evaluated in A2780, A2780cis, SK-OV-3 and OVCAR-3 cell cultures. As it is shown in Figure 2a, the level of SNAIL 1 protein proved to be significantly higher than level of SNAIL 2 in A2780cis, SK-OV-3 and OVCAR-3 but not in the A2780 cell line, which, in contrast, was characterized by the highest level of SNAIL 2. What is more, SNAIL 1 protein level was the highest in the SK-OV-3 and in A2780cis cell lines. Almost identical relations could be observed on the mRNA level of *SNAI1* and *SNAI2* genes in all tested cell lines (Figure 2b). The expression of *SNAI1* proved to be significantly higher than *SNAI2* in A2780cis, SK-OV-3 and OVCAR-3, but not in A2780 cells. *SNAI1* expression was the highest in SK-OV-3 and A2780cis cell lines, while the expression of *SNAI2* was the highest in A2780 cell line.

### 2.3. The Basal Surface Level of E-Cadherin and N-Cadherin on Ovarian Cancer Cell Lines

**Figure 3:**
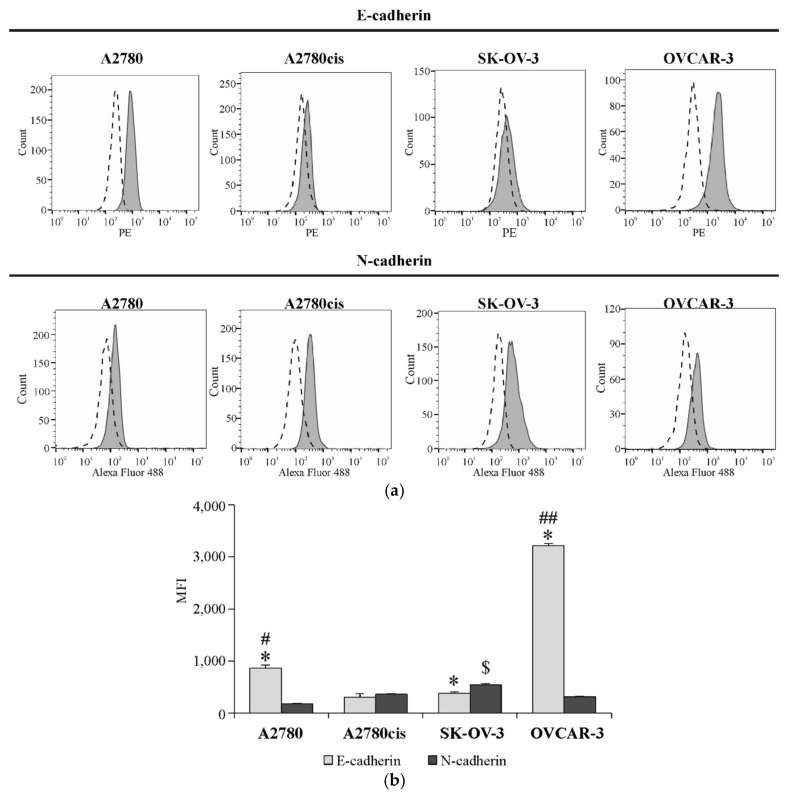
The basal level of E-cadherin and N-cadherin in A2780, A2780cis, SK-OV-3 and OVCAR-3 cell lines. Surface expression of both cadherins was determined by staining cells with antibodies, targeting either E-cadherin (Abs conjugated with PE fluorochrome) or N-cadherin (Abs conjugated with Alexa Fluor 488 fluorochrome) and measuring the intensity of fluorescence with the use of flow cytometry. (**a**) Representative histograms of E-cadherin and N-cadherin expression (grey graph) in regard to appropriate isotype controls (dashed line graph) are shown. (**b**) The acquired data are presented as the mean medium fluorescence intensity (MFI) ± SD from four independent experiments (*n* = 4). * Statistically significant difference in E-cadherin and N-cadherin level: E-cadherin vs. N-cadherin in A2780, A2780, SK-OV-3 or OVCAR-3 cell line, *p* ≤ 0.03 (Mann–Whitney *U* test test). # Statistically significant difference in E-cadherin level: A2780 vs. A2780cis or SK-OV-3 *p* ≤ 0.03 (Mann–Whitney *U* test) ## Statistically significant difference in E-cadherin level: OVCAR-3 vs. A2780 or A2780cis or SK-OV-3, *p* ≤ 0.03 (Mann–Whitney *U* test). $ Statistically significant difference in N-cadherin level: SK-OV-3 vs. A2780 or A2780cis or OVCAR-3, *p* ≤ 0.03 (Mann–Whitney *U* test).

We have determined the basal level of E-cadherin and N-cadherin proteins on the surface of A2780, A2780cis, SK-OV-3 and OVCAR-3 cells. The obtained data (Figure 3a,b) indicate that the level of E-cadherin was significantly higher in OVCAR-3 and A2780 cell lines than in other tested cell lines, while the level of N-cadherin was the highest in SK-OV-3 cells. What is more, considerable differences between both proteins’ expression were noticed in almost every tested cell line. As shown in Figure 3a,b, the level of E-cadherin was up to 5 and 10 times higher than the level of N-cadherin in A2780 an OVCAR-3 cells, respectively. On the other hand, N-cadherin expression was higher than E-cadherin expression in SK-OV-3 cell line, while the amount of these proteins was approximately similar in A2780cis cells.

### 2.4. Profiling the Activity of 43 Proteins in Ovarian Cancer Cell Lines. Determination of Selected Proteins Level and Activity

**Figure 4:**
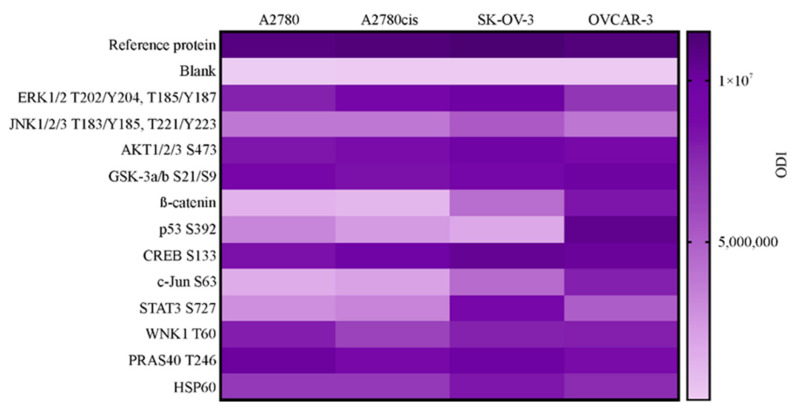
The phosphorylation level of 12 chosen signaling proteins in A2780, A2780cis, SK-OV-3 and OVCAR-3 cell lines. Cultured cells were harvested, lysed and the level of phosphorylated proteins was determined with Proteome Profiler Human Phospho-Kinase Array kit. All samples were made in duplicate. The acquired dots were quantified by densitometric analysis and data are presented as a heat map of the optical density intensity (ODI) of the area under each band’s peak.

In the following stage of the study, we profiled the activity of 43 proteins in A2780, A2780cis, SK-OV-3 and OVCAR-3 cell lines using a commercial human proteome profiler kit. The principle of this kit is based on the comparison of optical density intensity (ODI) of phosphorylated proteins to the reference protein. Firstly, we have chosen the proteins with ODI, accounting for 30% or more of reference protein’s ODI value in at least one of the tested cell lines. As a result, we have selected the 20 most active proteins, which underwent further selection. Secondly, we have narrowed the choice of proteins and decided to select only those in which ODI amounted for 50% or more of the reference protein’s ODI value in at least one of the tested cell lines. As shown in Figure 4, the 12 most active proteins in A2780, A2780cis, SK-OV-3 and OVCAR-3 cell lines consisted of ERK 1/2, JNK 1/2/3, AKT 1/2/3, GSK-3 a/b, β-catenin, p53, CREB, c-JUN, STAT3, WNK1, PRAS40 and HSP60. All profiled proteins and their ODI values are presented in Table S1. For subsequent study, we have chosen three proteins, ERK 1/2, AKT and STAT3, as their phosphorylation level is one of the highest and they are known to participate in intracellular signaling pathways involved in cancer cells’ growth, survival and response to cytotoxic agents [23,24,25].

**Figure 5:**
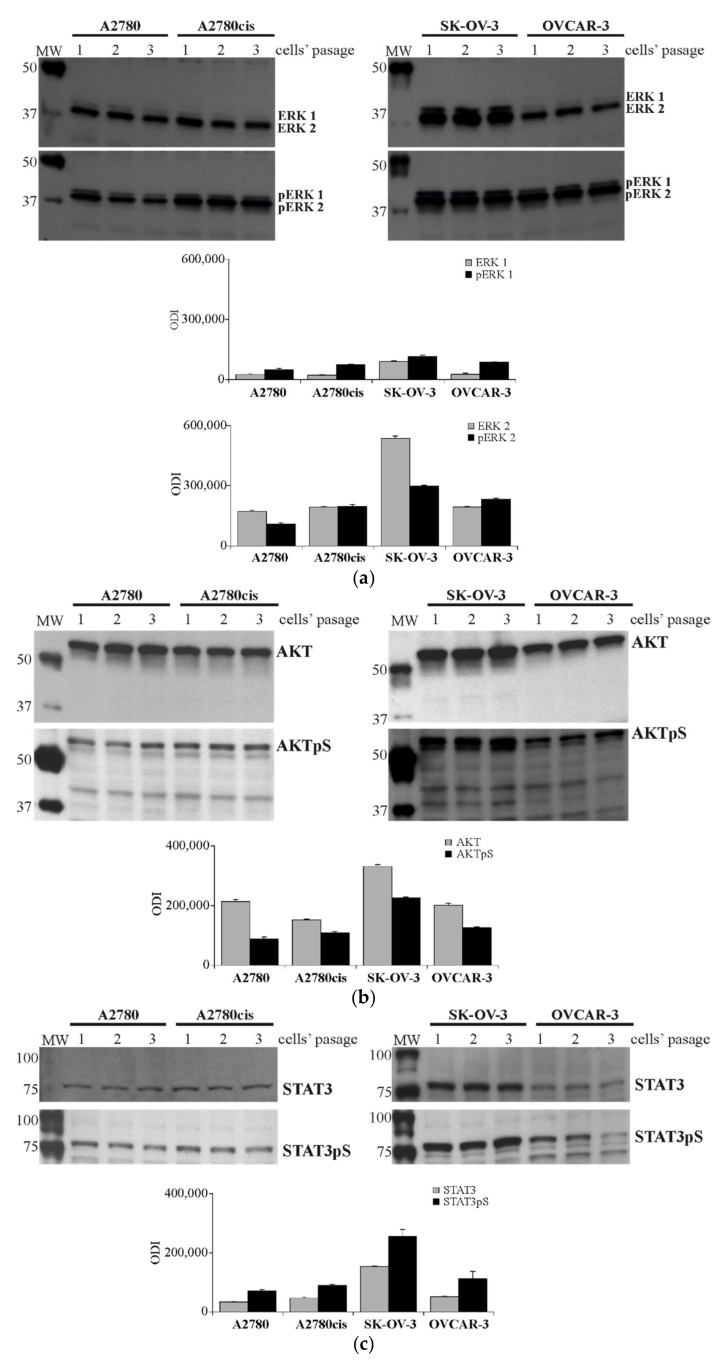

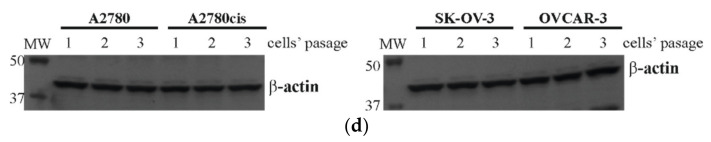
The total and phosphorylation level of ERK 1/2, AKT and STAT3 signaling proteins in A2780, A2780cis, SK-OV-3 and OVCAR-3 cell lines. Cultured cells were harvested, lysed and the level of total and phosphorylated proteins was determined with Immunoblotting-ECL method. Representative immunoblots of ERK 1/2 (**a**), AKT (**b**), STAT3 (**c**). Additionally, β-actin (**d**) was used as a loading control for all samples. Bands were quantified by densitometric analysis and data are presented as the optical density intensity (ODI) of the area under each band’s peak ± SD from three independent experiments (*n* = 3).

The level of total and phosphorylated forms of ERK 1/2, AKT and STAT3 proteins in all tested cell lines was evaluated with an independent immunoblotting-ECL assay before subsequent analysis. The antibodies were specifically targeted against total and phosphorylated forms on threonine, and tyrosine residues (ERK 1/2 pThr202/Tyr204) or serine residues (AKT Ser473, STAT3 Ser727) were used to visualize the level of these proteins. As shown in Figure 5a–c, the obtained results confirmed that phosphorylation of pERK 1/2, AKT pS and STAT3 pS was high in all tested cell lines, being the highest in the SK-OV-3 cell line and the lowest in the A2780 cell line.

### 2.5. The Effect of Inhibiting ERK 1/2, AKT and STAT3 Signaling Proteins Activity on the Expression of SNAIL 1 and SNAIL 2 in Ovarian Cancer Cell Lines

**Figure 6:**
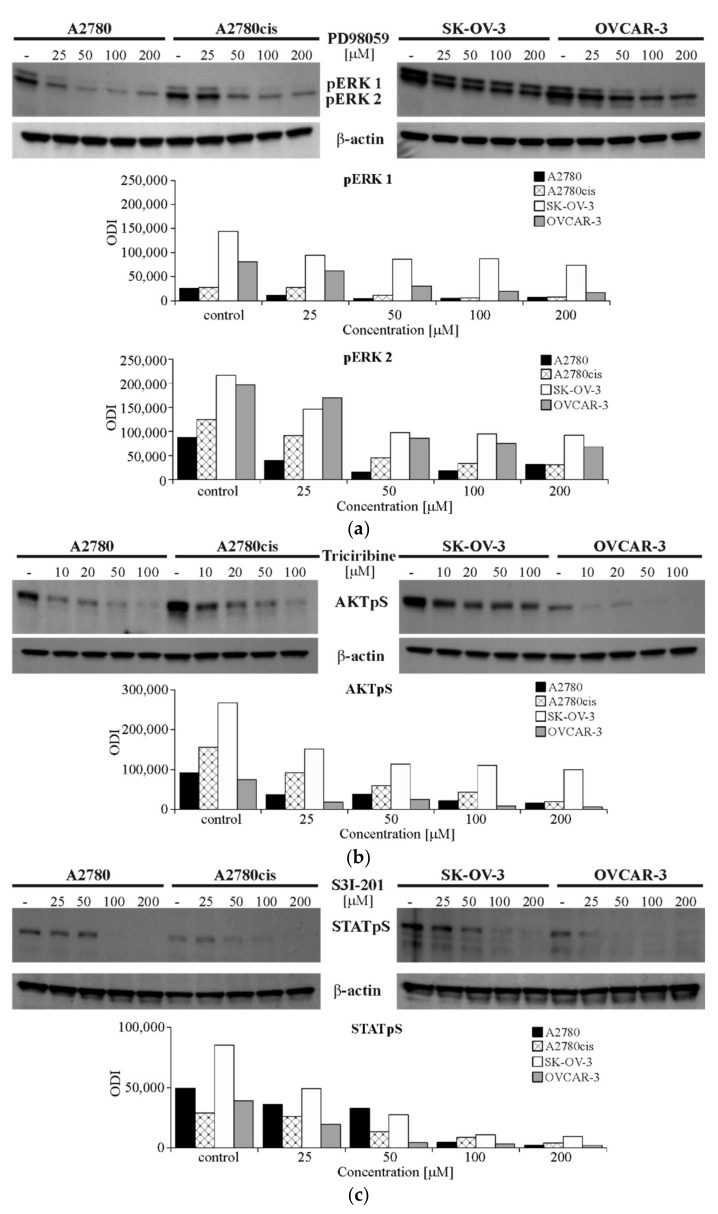

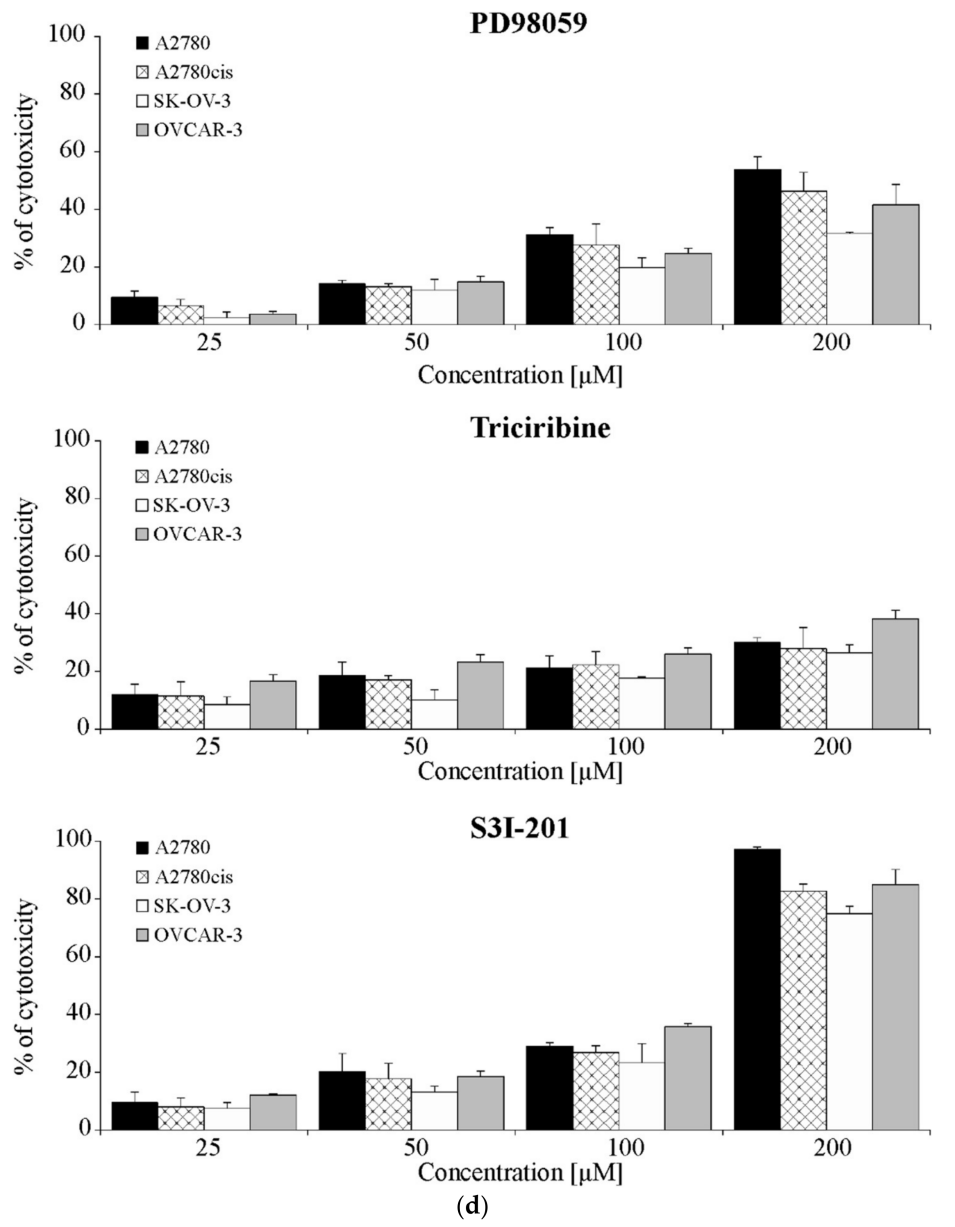
Determination of effective concentration of PD98059, Triciribine and S3I-201. All cell lines were cultured with PD98059 [25–200 µM] or Triciribine (10–200 µM) or S3I-201 (25–200 µM) or not (control) for 48 h. (**a**–**c**) The phosphorylation level of signaling proteins was determined with Immunoblotting-ECL method. Representative immunoblots of ERK 1/2 (**a**) or AKT (**b**) or STAT3 (**c**) phosphorylation, along with β-actin level are presented. Bands were quantified by densitometric analysis and data are presented as the optical density intensity (ODI) of the area under each band’s peak two independent experiments (*n* = 2). (**d**) Cell viability was determined with MTT assay and is presented as the mean percent of cytotoxicity ± SD calculated from three independent experiments (*n* = 3).

In order to evaluate the possible impact of ERK 1/2, AKT and STAT3 in the regulation of SNAIL 1 and SNAIL 2 protein level and mRNA level, the pharmacological inhibitors of these proteins’ activation were used. The authors have chosen PD98059 (inhibitor of MEK 1/2 activation—the upstream kinases required for ERK 1/2 activation), Triciribine (AKT inhibitor) and S3I-201 (STAT3 inhibitor). These are most commonly used compounds with confirmed specificity toward targeted proteins, as indicated by the manufacturer’s data sheet. In the first step, the effective concentrations of PD98059, Triciribine and S3I-201 were determined for each cell line. The choice of effective concentration of each compound was based on their ability to significantly reduce appropriate protein’s phosphorylation (Immunobloting-ECL), as well as on their cytotoxicity toward ovarian cancer cells (MTT assay). The concentrations selected for further study were proved to significantly reduce protein’s phosphorylation by at least 50% (Figure 6a–c). At the same time, their cytotoxicity did not exceed 30% of dead ovarian cancer cells (Figure 6d). The impact of DMSO (solvent of tested inhibitors) on cell lines’ viability, at the highest concentration of inhbitors used in the course of this experiment (approximately 2%) reached cytotoxicity of 8%, 5%, 8% and 7% for A2780, A2780cis, SK-OV-3 and OVCAR-3, respectively. The final effective concentrations of every compound, chosen for subsequent study, were as follows: PD98059—50 μM (all cell lines); Triciribine 50 μM (SK-OV-3 and OVCAR-3) and 100 μM (A2780 and A2780cis); S3I-201 50 μM (OVCAR-3) and 100 μM (A2780, A2780cis and SK-OV-3). The cytotoxicity of DMSO, at these final concentrations, amounted for 2.9% (A2780), 0.6% (A2780cis), 1.1% (SK-OV-3) and 2.6% (OVCAR-3) and was not the main determining factor of cells’ viability.

**Figure 7:**
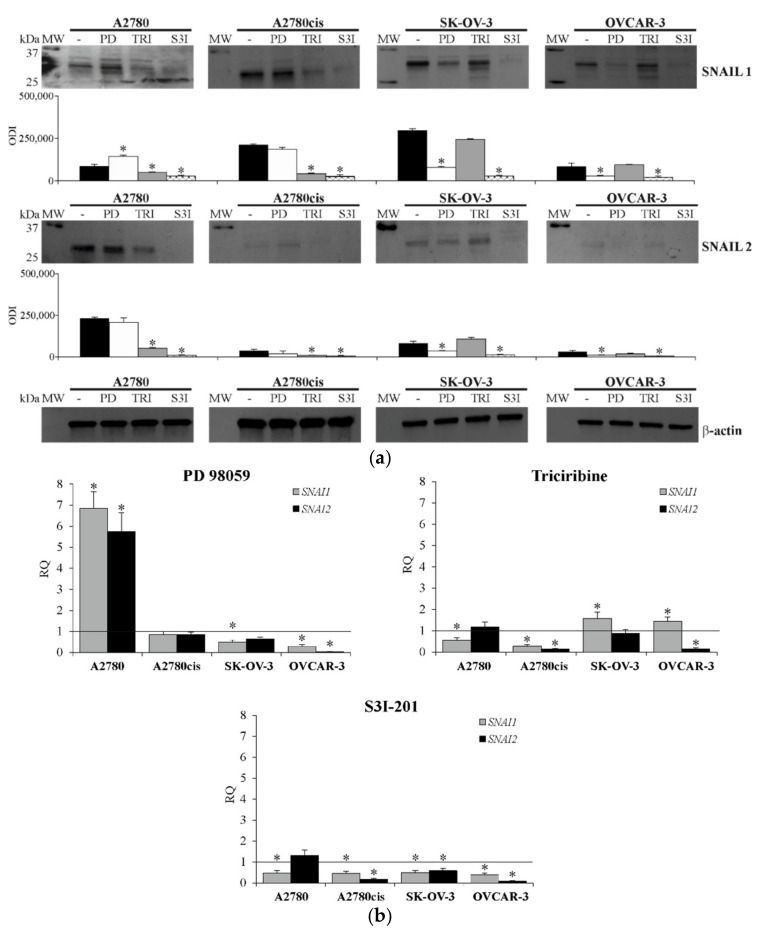
The expression of SNAIL 1 and SNAIL 2 in A2780, A2780cis, SK-OV-3 and OVCAR-3 cell lines treated with inhibitors. All cell lines were cultured with PD98059 (PD) (50 µM) or Triciribine (TRI) (50 or 100 µM) or S3I-201 (S3I) (50 or 100 µM) or not (control) for 48 h. (**a**) The level of SNAIL 1 and SNAIL 2 was determined with immunobloting-ECL method. Representative immunoblots of SNAIL 1 and SNAIL 2 protein level are presented. Additionally, β-actin was used as a loading control for all samples. Bands were quantified by densitometric analysis and data are presented as the optical density intensity (ODI) of the area under each band’s peak ± SD from five independent experiments (*n* = 5). * Statistically significant change in SNAIL 1 or SNAIL 2 level: PD98059 or Triciribine or S3I-201 vs. control, *p* ≤ 0.04 (Wilcoxon’s singed rank test). (**b**) The expression of *SNAI1* and *SNAI2* genes was determined with real-time PCR assay. Data are presented as mean RQ from four independent experiments (*n* = 4). RQ (relative quantification) represent the fold change of expression in relation to untreated cells (control) and it was calculated using RQ Data Assist software v1.2 (Applied Biosystems). * Statistically significant change in *SNAI1* or *SNAI2* expression: PD98059 or Triciribine or S3I-201 vs. control, *p* ≤ 0.01 (*t*-test).

Exposing ovarian cancer cell lines to ERK 1/2, AKT and STAT3 inhibitors for 48 h, resulted in a significant change in SNAIL 1 and SNAIL 2 expression in all ovarian cancer cell lines, however, this effect depended on the kind of inhibited signaling protein, as well as on the type of cell line. Obtained data (Figure 7a) clearly indicate that blocking the activity of ERK 1/2 resulted in a significant reduction in SNAIL 1 and SNAIL 2 level in SK-OV-3 and OVCAR-3 cell lines, whereas it significantly increased the level of SNAIL 1 (but not SNAIL 2) in A2780 cell line and did not affect the level of these transcription factors in A2780cis cell line. On the other hand, inhibiting the activity of AKT significantly decreased amount of SNAIL 1 and SNAIL 2 in A2780 and A2780cis cell lines, whilst not affecting the level of both evaluated proteins in SK-OV-3 and OVCAR-3 cell lines. The impact of STAT3 activity inhibition was the most interesting one, since it significantly reduced the level of SNAIL 1 and SNAIL 2 in all tested cell lines.

Simultaneous evaluation of *SNAI1* and *SNAI2* genes expression (Figure 7b) showed that inhibiting the activity of ERK 1/2, AKT and STAT3 signaling proteins significantly affected *SNAI1* and *SNAI2* mRNA level. It is worth mentioning that the acquired data are mostly on par with the changes observed in SNAIL 1 and SNAIL 2 protein level. The obtained results indicate that blocking the activity of ERK 1/2 led to a significant drop in the expression of *SNAI1* in the SK-OV-3 cell line, as well as both *SNAI1* and *SNAI2* in the OVCAR-3 cell line. On the other hand, it significantly enhanced the expression of both genes in A2780 cell line, whereas no significant change was observed in the A2780cis cell line. What is more, inhibiting the activity of AKT resulted in a significant drop in *SNAI1* expression in A2780 cells and both genes in the A2780cis cell line. A significant increase in *SNAI1* could be observed in SK-OV-3 and OVCAR-3 cell lines, however, a noticeable decrease in *SNAI2* level was present in the OVCAR-3 cell line. Once again, inhibiting STAT3 activity resulted in a significant drop in *SNAI1* and *SNAI2* expression in all tested cell lines.

### 2.6. The Level of E-Cadherin and N-Cadherin in Ovarian Cancer Cell Lines Exposed to the Inhibitors of ERK 1/2, AKT and STAT3

In the next part of the research, we wanted to evaluate in what way the changes in SNAIL 1 and SNAIL 2 expression, caused by inhibitors of significant proteins, affected the phenotype of tested cell lines. Therefore, we analyzed the surface expression of E-cadherin and N-cadherin in A2780, A2780cis, SK-OV-3 and OVCAR-3 cell lines exposed to ERK 1/2, AKT and STAT3 inhibitors for 48 h. Generally, the obtained data indicate that the level of both cadherins depended on the kind of inhibited signaling protein, as well as on the type of cell line, and, in most cases, it was related to the expression of SNAIL 1 and SNAIL 2 in particular cell line.

**Figure 8:**
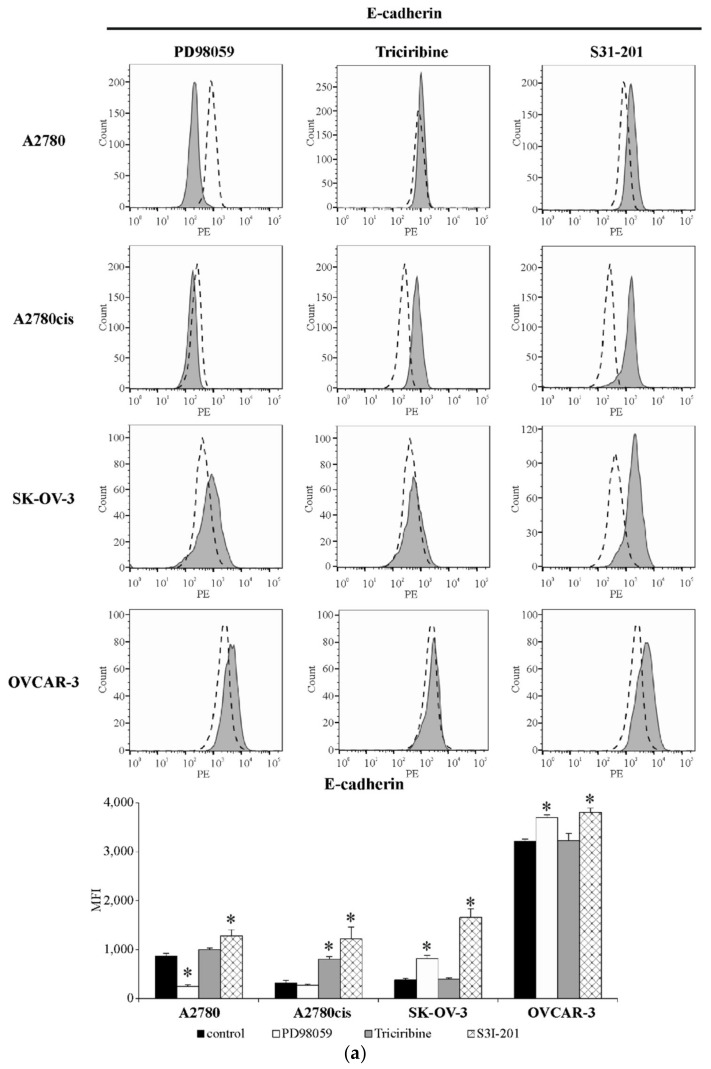

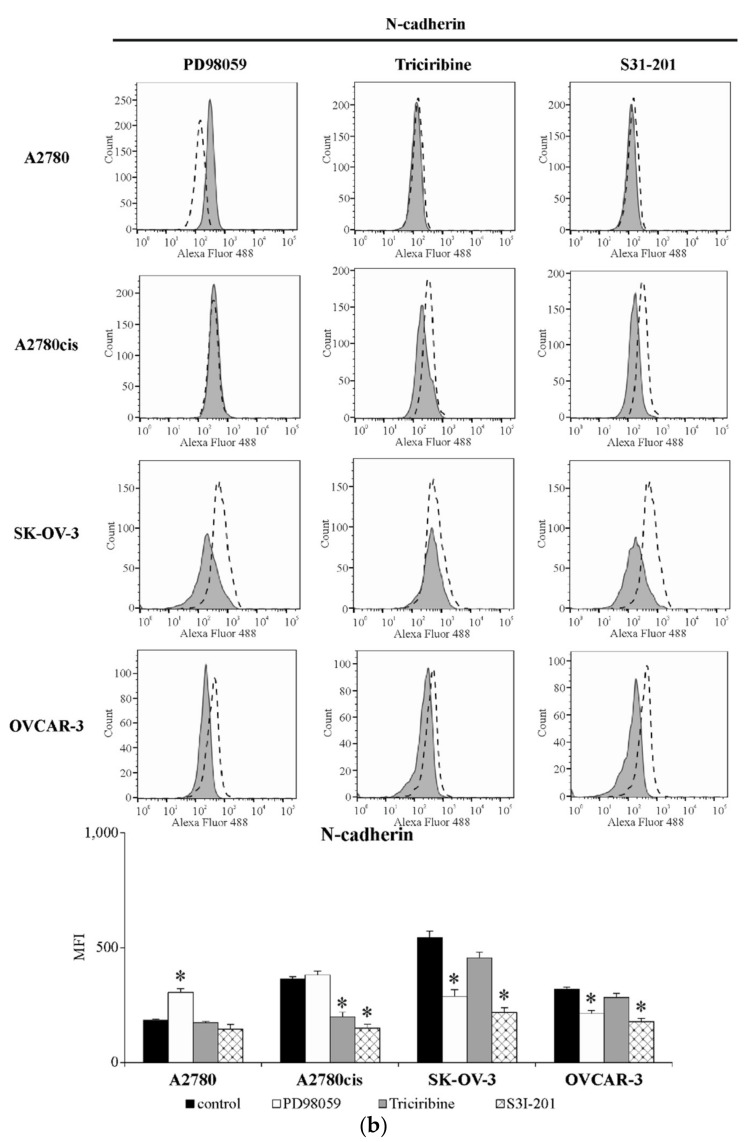
The level of E-cadherin and N-cadherin in A2780, A2780cis, SK-OV-3 and OVCAR-3 cells treated with inhibitors. All cell lines were cultured with PD98059 (50 µM) or Triciribine (50 or 100 µM) or S3I-201 (50 or 100 µM) or not (control) for 48 h. Surface expression of both cadherins was determined by staining cells with antibodies, targeting either E-cadherin (Abs conjugated with PE fluorochrome) or N-cadherin (Abs conjugated with Alexa Fluor 488 fluorochrome) and measuring the intensity of fluorescence with the use of flow cytometry. Representative histograms of E-cadherin (**a**) or N-cadherin (**b**) expression in cells treated with PD98059 or Triciribine or S3I-201 (grey graph) in regard to the E-cadherin or N-cadherin expression in untreated cells (dashed line graph) are shown. The acquired data are presented as the mean of median fluorescence intensity (MFI) ± SD from five independent experiments (*n* = 5). * Statistically significant change in E-cadherin or N-cadherin level: PD98059 or Triciribine or S3I-201 vs. control, *p* ≤ 0.03 (Wilcoxon’s singed rank test).

The results presented in Figure 8a,b indicate that inhibiting ERK 1/2 activity led to a significant drop in E-cadherin level in A2780 cells with a simultaneous noteworthy increase in N-cadherin level in both of these cell lines. Conversely, the significant growth in E-cadherin and drop in N-cadherin expression was observed in SK-OV-3 and OVCAR-3 cell lines. On the other hand, blocking the activity of AKT significantly enhanced E-cadherin and lowered N-cadherin level in A2780cis cells, but did not affect the expression of both proteins in A2780, SK-OV-3 and OVCAR-3 cell lines. Interestingly, inhibiting STAT3 activity resulted in a significant increase in E-cadherin expression in all tested cell lines, accompanied by a significant drop in N-cadherin expression in A2780cis, SK-OV-3 and OVCAR-3, but not A2780, cells.

### 2.7. The Effect of Inhibiting ERK 1/2, AKT and STAT3 Signaling Proteins Activity on A2780, A2780cis, SK-OV-3 and OVCAR-3 Cells Response to Cisplatin Treatment

**Figure 9:**
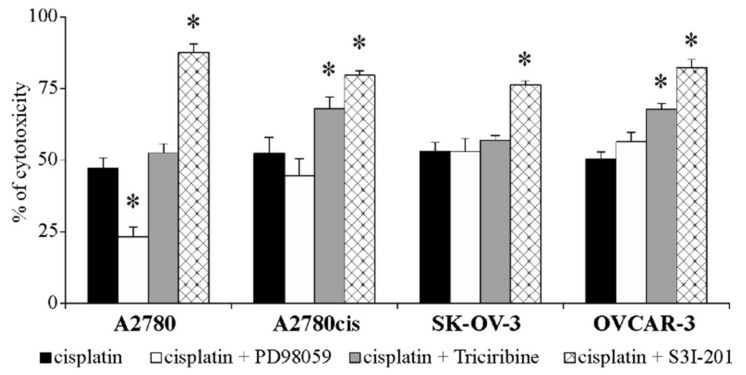
The viability of A2780, A2780cis, SK-OV-3 and OVCAR-3 cells treated with the combination of cisplatin and inhibitors. All cell lines were cultured with cisplatin (6 or 20 or 32 or 41 µM) alone or in combination with PD98059 (50 µM) or Triciribine (50 or 100 µM) or S3I-201 (50 or 100 µM) or left untreated (control) for 48 h. Initially, all cell lines were pre-treated with appropriate inhibitors for 1 h before addition of cisplatin. Cell viability was determined with MTT assay and is presented as the mean % of cytotoxicity ± SD from five independent experiments (*n* = 5). * Statistically significant change in cell viability: PD98059 + cisplatin or Triciribine + cisplatin or S3I-201 + cisplatin vs. cisplatin, *p* ≤ 0.04 (Wilcoxon’s singed rank test).

In the last stage of this study, we wanted to determine whether changes in SNAIL 1 and SNAIL 2 expression, caused by treating ovarian cancer cell lines with PD98059, Triciribine and S3I-201, in any way alter the cells response to cisplatin. Therefore, we exposed all tested cell lines to the combination of cisplatin (at the concentration equal to EC_50_ value for each cell line) and selected inhibitors for 48 h. The obtained results indicate that the addition of ERK 1/2 or AKT or STAT3 inhibitors to cisplatin-only treatment, significantly affected cell sensitivity/resistance to this drug and that effect depended on the kind of inhibited signaling protein, and on the type of cell line. As it is shown in Figure 9, blocking ERK 1/2 activity resulted in significantly increased A2780 cell viability but, simultaneously, no changes in cisplatin cytotoxicity to A2780cis, SK-OV-3 and OVCAR-3 cells were observed. In contrast, inhibiting AKT activity significantly decreased A2780cis and OVCAR-3 cells’ sensitivity to cisplatin, while having no impact on A2780 and SK-OV-3 cells’ viability. Interestingly, impairing STAT3 activity resulted in significantly increased cisplatin toxicity to all tested cell lines.

## 3. Discussion

Cancer drug resistance is frequently accompanied by EMT in diverse cancers, including ovarian [8,9,10,11]. The involvement of SNAIL 1 and SNAIL 2 in ovarian cancer cells chemoresistance, EMT and disease progression has been already established and well described [13,26,27,28]. However, the mechanism of the parallel regulation of chemoresistance and EMT in ovarian cancer cells is still not well recognized. Therefore, the main goal of this study was to assess the possible impact of SNAIL 1 and SNAIL 2 expression, regulated by three selected signaling proteins on EMT markers (cadherins) level in various ovarian cancer cell lines and their resistance to cisplatin. To the best of our knowledge, this kind of comparative analysis (few signaling proteins, various ovarian cancer cell lines), performed in a single set of experiments, has never been reported before.

The ovarian cancer cell lines used in this study proved to differ substantially in sensitivity to cisplatin. Our data have shown that SK-OV-3 and A2780cis cell lines are characterized with the highest cisplatin resistance, which is up to six-fold greater than the resistance of A2780 cells—the most cisplatin-sensitive cell line. Similar observations regarding cisplatin sensitivity/resistance have been made by other investigators [29,30]. At this point, it is essential to emphasize that the A2780cis cell line differs from SK-OV-3 and OVCAR-3 cells in terms of chemoresistance acquisition. According to cell line datasheets, A2780cis cells have an acquired chemoresistance, developed artificially by chronic exposure to cisplatin. SK-OV-3 and OVCAR-3 cell lines, on the other hand, underwent epigenetic changes, allowing them to disseminate from primary tumor to ascites, thus developing the chemoresistance termed as intrinsic. This may significantly affect various cells’ properties. Moreover, it is worth to mention that presented cell lines also differ in place of origin, with A2780 and A2780cis cells originating from ovary tissue, while SK-OV-3 and OVCAR-3 cells originating from ascites [31]. This may be an important factor, determining cells’ metastatic potential. In our previous study, we reported that both SK-OV-3 and OVCAR-3 cells have the ability to migrate through matrigels [32].

The evaluation of SNAIL 1 and SNAIL 2 expression in tested ovarian cancer cell lines indicate that there are significant differences between particular cell lines in terms of the level of these transcription factors. The fact that the protein and mRNA levels of SNAIL 1 are the highest in chemoresistant A2780cis and SK-OV-3 cell lines, while SNAIL 2 is the highest in cthe isplatin-sensitive, yet dynamically growing, A2780 cell line, may suggest that SNAIL 1 but not SNAIL 2 is related with ovarian cancer cell resistance to chemotherapeutic agent. This seems to confirm the observations made by Sonego et al. [33], which indicated that the regulation of SNAIL 1 protein stability affected cisplatin resistance in SK-OV-3 and OVCAR-3 cell lines. Similarly, the involvement of SNAIL 1 in cisplatin resistance was also described in cell lines of other cancer types, such as lung adenocarcinoma, as well as head and neck squamous cancer [34,35]. The possible reason for difference in the involvement of both transcription factors in cisplatin resistance can be explained by the fact that one of the specific targets for SNAIL 1, but not SNAIL 2 or SNAIL 3, is phosphatase and tensin homolog deleted in chromosome 10 (PTEN) [20]. PTEN is a well-known tumor suppressor involved in the regulation of PI3K/AKT signaling pathway, cell cycle and induction of apoptosis. The repression of PTEN activity by SNAIL 1 contributes to cells’ resistance to apoptosis, as well as the development of resistance to platinum compounds [36,37].

In order to define the epithelial or mesenchymal phenotype of each cell line, we analyzed the basal expression of well-known EMT markers, like E-cadherin and N-cadherin [38] on the surface of ovarian cancer cells used in this study. The loss of E-cadherin with simultaneous up-regulation of N-cadherin is called a “cadherin switch” and it is driven primarily by transcription factors, such as SNAIL, TWIST or ZEB, which negatively modulate E-cadherin expression. This phenomenon is defined as a key feature of the EMT process [39].

The obtained results indicate that OVCAR-3 and A2780 cells are characterized by a high level of E-cadherin, whereas the highest N-cadherin expression was present in SK-OV-3 and A2780cis cell lines. A study performed by Yang et al. [40], with the use of A2780 and A2780/DDP (cisplatin-resistant) cell lines, also characterized A2780 as E-cadherin-high/N-cadherin-low and A2780/DDP as E-cadherin-low/N-cadherin-high cells. Similarly, Rosso et al. [41] noticed low E-cadherin/high N-cadherin expression in the SK-OV-3 cell line, which confirms our own findings. Moreover, E- and N-cadherin reflects these cells’ migration capacity. As was shown by Ruibin et al. [42] and Wang et al. [43], A2780 cells were less motile and invasive than chemoresistant A2780-M cells (derived from the A2780 cell line) and SK-OV-3 cells. Furthermore, in our own study, we have shown that SK-OV-3 cells can migrate through a matrigel to a much higher extent that OVCAR-3 cells [37]. Therefore, on the basis of ours and other results, it could be suggested that highly cisplatin-resistant SK-OV-3 and A2780cis cells have a higher invasive potential than other tested cell lines. It is also important to underline that those two cell lines are also characterized by the highest SNAIL 1 expression, indicating that its elevated level is related to increased EMT markers’ level and the chemoresistance of ovarian cancer cells.

In further study, we have decided to focus on the involvement of three effector proteins (ERK 1/2, AKT and STAT3) in the SNAILs-dependent induction of EMT markers (assessment of cadherins level), as well as in the chemoresistance of ovarian cancer cells. We decided to choose ERK 1/2, AKT and STAT3, since their expression and activity are usually enhanced in ovarian cancer [23,44,45]. Moreover, they are involved in the induction of ovarian cancer cells EMT [46,47,48], as well as in their response to chemioterapeutic drugs [49,50,51]. The strategy of inhibiting ERK 1/2, AKT and STAT3 activity is extensively studied in various cancers, as these proteins are believed to be essential in overcoming the invasiveness and chemoresistance of cancer cells [12,14,24,45,52,53]. Nevertheless, the impact of selected proteins on SNAIL 1 and SNAIL 2 expression, and, in consequence, their modulation of ovarian cancer’s metastatic potential and chemoresistance in a SNAIL-dependent manner, is still unclear. It is also worth noting that we were aiming at the proteins, in which activity is high but, at the same time, differs among tested cell lines (such as STAT3 and to the less extent ERK 1/2). As cell lines used in this study are characterized by different origins and properties regarding cisplatin resistance and invasive potential, the importance of selected proteins’ activity could vary for each cell line. We wanted to verify whether the observed differences affect these proteins’ impact the expression of SNAIL transcription factors. Our study has shown that the inhibition of ERK 1/2, AKT and STAT3 activity significantly affected the expression of SNAIL 1 and SNAIL 2 as well as the levels of E-cadherin and N-cadherin in various ovarian cancer cell lines. Moreover, inhibiting the activity of selected signaling proteins also significantly changed cells’ sensitivity to cisplatin. However, the final effect of these modulations depended on the type of signaling protein and varied between different types of ovarian cancer cell lines.

We have observed that impairment of ERK 1/2 signaling resulted in a drop in SNAIL 1 and SNAIL 2 expression in SK-OV-3 and OVCAR-3 cell lines (originating from ascites), along with the enhanced level of E-cadherin and diminished level of N-cadherin. On the other hand, no significant alteration in the level of both SNAILs as well as cadherins was observed in cisplatin-resistant A2780 cells (originating from ovary tissue). Interestingly, a significant increase in SNAIL 1 level was observed in cisplatin-sensitive, tissue-derived A2780 cells, accompanied by a drop in E-cadherin expression and up-regulated N-cadherin. Furthermore, blocking ERK 1/2 activity increased resistance to cisplatin in A2780 cells, but it did not affect the A2780cis, SK-OV-3 and OVCAR-3 cells’ response to this chemotherapeutic agent. Since there are not as many comparative reports regarding ERK 1/2–SNAIL interactions in ovarian cancer model, it is hard to directly evaluate and compare our data with others. Nevertheless, there are some very interesting reports which may shed some light on this assumption. For instance, Latifi et al. [54] reported that blocking ERK 1/2 signaling suppressed SNAIL-induced EMT in cisplatin-resistant OVCA-433 cell line, originating from ascites. Moreover, Cheng et al. [55] pointed at a significant downregulation in SNAIL and N-cadherin in the SK-OV-3 cell line, as a result of the drop in ERK 1/2 activity. What is more, Sawada et al. [56] has shown that reduced activity of ERK 1/2, related to the loss of E-cadherin in A2780, SK-OV-3 and OVCAR-5 cells, indicating that the activation of these kinases is cadherin-dependent. The contribution of these kinases to cancer progression and chemoresistance has been described in many types of cancer, including ovarian cancer [57], and some of the studies suggest that this mechanism of action is SNAIL-dependent [58]; however, this has not been fully confirmed to daye. Based on the presented data, it is possible to assume that the expression of SNAIL transcription factors is at least partially regulated by ERK 1/2 activity. Considering the fact that the presented cell lines differ substantially in the basal level of E-cadherin and N-cadherin expression (as described above), the site of their origin, and their resistance to cisplatin, it may be suggested that the involvement of ERK 1/2 proteins in the regulation of SNAIL 1 and SNAIL 2 expression is tightly related to EMT marker level and the intrinsic resistance of ovarian cancer cells to chemotherapeutic agents.

Blocking AKT activity, on the other hand, significantly reduced SNAIL 1 and SNAIL 2 level in ovary-tissue-derived A2780 and A2780cis cell lines, regardless of their resistance to cisplatin, while it had no effect on these transcription factors in SK-OV-3 and OVCAR-3 cells, which are resistant to cisplatin and originate from ascites. However, the upregulation of E-cadherin and down-regulation of N-cadherin was noticed only in A2780cis cells. Interestingly, the significant increase in sensitivity to cisplatin was observed in chemoresistant A2780cis and OVCAR-3 cell lines. It has been shown that the downregulation of AKT activity led to a drop in SNAIL expression and the invasiveness of A2780 cells [47]. Interestingly, in contradiction to our results, this phenomenon has also been observed in SK-OV-3, OVCA-433, OVCAR-4 and OVCAR-5, all of which are cisplatin-resistant and originate from ascites. However, it is important to point out that most of these studies have described a different approach in affecting the PI3K/AKT signaling pathway and focused on targeting ATM serine threonine protein kinase [47], Wip1 phosphatase [59] or chemokine receptor CXCR7 [46] rather than AKT itself, which could be the reason why we and others have observed different results. Moreover, the study of Deng et al. [60] seems to confirm our own observation, pointing out that inhibition of the PI3K/AKT signaling pathway inhibited EMT and reduced the invasiveness of A2780cis cells, making them more sensitive to cisplatin. Taken together, our and others data suggest that AKT-dependent downregulation of SNAIL 1 and SNAIL 2 expression in ovarian cancer cells is not necessarily determined by their resistance to cisplatin but rather by their place of origin. However, the reduction in EMT markers and sensitization to cisplatin, due to impaired AKT activity, was mainly observed in chemoresistant cells. Thus, this may indicate that the mechanism of this action may not only depend on SNAIL expression, as was suggested by Liu et al. [61], who has shown that the blocking activity of AKT inhibited the EMT and invasiveness of SK-OV-3 and OVCAR-3 cells in a TWIST-dependent manner.

Our data indicated that neither resistance to cisplatin nor basal migration potential (along with the site of origin) mattered in the case of STAT3 activity, the inhibition of which significantly reduced SNAIL 1 and SNAIL 2 expression, altered cadherin level and sensitized all tested ovarian cancer cell lines to cisplatin. Similar observations were made by Ma et al. [62], who has shown that A2780 and SK-OV-3 cells transfected with STAT3 decoy oligodeoxynucleotide complexes were characterized by no STAT3 activity, SNAIL downregulation and the inhibition of cell invasion. Likewise, Liang et al. [63] indicated the downregulation of SNAIL transcription factors, E-cadherin and Vimentin in SK-OV-3 and OVCA429 transfects with silenced STAT3. Moreover, in the same study, they also described that the lack of STAT3 activity promoted sensitivity to cisplatin. To date, there are no available data showing the impact of direct STAT3 inhibitors on SNAIL 1 and SNAIL 2 expression and/or the induction of EMT in various ovarian cancer cell lines. Nevertheless, the compounds directly inhibiting STAT3 significantly reduced SNAIL expression and diminished EMT in osteosarcoma cells [64] and colorectal cancer [65]. The discussed results indicate the universal character of STAT3 in the maintenance of both SNAIL 1 and SNAIL 2 expression in ovarian cancer cell lines, independently of cells’ origin, basal invasive potential or their resistance to cisplatin.

The presented results, although mainly observational, suggest that SNAIL 1 and SNAIL 2 have a potentially important role in the regulation of ovarian cancer chemoresistance. We believe that our data are not without merit, as this kind of screening has never been reported before for ovarian cancer. Moreover, this research points at the necessity of using possibly the most diverse ovarian cancer model, since the obtained data may heavily depend on the type of cancer cell line. Thus, it is crucial to at least attempt to reflect the high heterogeneity of ovarian cancer to see the full spectrum of possible interactions.

## 4. Materials and Methods

### 4.1. Reagents and Antibodies

Trypsin 0.05% EDTA solution, RPMI 1640 medium with 2 mM l-glutamine and 1 mM sodium pyruvate, CL-Xposure film and Dulbecco’s phosphate buffered saline (D-PBS) were purchased from Gibco (Thermo Fisher Scientific, Foster City, CA, USA). Heat-inactivated fetal bovine serum (FBS) was obtained from EURx (Gdansk, Poland). 3-(4,5-dimethylthiazol-2-yl)-2,5-diphenyltetrazolium bromide (MTT), cis/diammineplatinum(II) dichloride, dimethyl sulfoxide (DMSO), PD98059, Triciribine, S3I-201, phenylmethylsulfonyl fluoride (PMSF), EGTA, Tris, Tween 20, bromophenyl blue, ethanol, 2-propanol and penicillin/streptomycin were purchased from Sigma-Aldrich (Merck, Germany). Proteome Profiler Human Phospho-Kinase Array was purchased from R&D biosystems (Minnepolis, CA, USA). RIPA lysis buffer, superBlock Blocking Buffer in TBS, 10 × Tris-glycine-SDS buffer, Halt protease and phosphatase inhibitor cocktail and ECL Western Blotting substrate kit were obtained from Thermo Fisher Scientific (USA). A DC protein assay kit, 10% SDS-PAGE mini-protean precast TGX gel, trans-blot turbo transfer pack PVDF and precision plus protein western C standard were obtained from BioRad (Hercules, CA, USA). TRIzol^®^ Reagent, TaqMan^®^ Universal PCR Master Mix, Maxima First Strand cDNA Synthesis Kit and TaqMan^®^ Gene Expression Assays for *β-actin*, *GAPDH*, *SNAI1* and *SNAI2* were bought from Life Technologies (Thermo Fisher Scientific, Foster City, CA, USA). Antibodies, mouse anti-SNAIL1 monoclonal IgG, rabbit anti-SNAIL2 monoclonal IgG, were purchased from Cell Signaling Technology (Danvers, MA, USA). Rabbit polyclonal IgG anti-AKT/PKB, rabbit monoclonal IgG anti-pAKT/PKB [Ser-473], mouse monoclonal IgG anti-STAT3, rabbit polyclonal IgG anti-pSTAT3 [Ser-727], HRP-conjugated goat anti-rabbit IgG (H+L), HRP-conjugated goat anti-mouse IgG (H+L), were purchased from Invitrogen (Thermo Fisher Scientific, Foster City, CA, USA). Mouse IgG anti-*β* actin antibody was purchased from Sigma-Aldrich (Merck, Germany). Mouse anti-human CD324 (E-cadherin) PE, mouse anti-human CD325 (N-cadherin) Alexa Fluor 488 and mouse IgG1 κ isotype control Alexa Fluor 488 were purchased from BD pharmingen (Franklin Lakes, NJ, USA). Mouse IgG1 κ isotype control PE was purchased from R&D biosystems (Minneapolis, CA, USA).

### 4.2. Cell Lines Culture and Treatment

Four cell lines were investigated in this study: A2780 and A2780cis cell lines (purchased from ECACC General cell collection (Salisbury, UK)), as well as SK-OV-3 and OVCAR-3 (purchased from ATCC (Manassas, VA, USA)). All cell lines are of epithelial origin and characterize with adherent growth in the form of a monolayer. A2780cis, SK-OV-3 and OVCAR-3 cell lines are classified as cisplatin-resistant, while the A2780 cell line is classified as cisplatin-sensitive. Moreover, A2780 and A2780cis originate from ovary tissue, whereas SK-OV-3 and OVCAR-3 were collected from ascites. Cells were cultured in RPMI 1640 medium with 2 mM L-glutamine, 1 mM sodium pyruvate, 10% FBS (20% of FBS in case of OVCAR-3 cells), as well as 100 U of penicillin and 10 mg of penicillin. Cells were passaged every 2–3 days by trypsinization (Trypsin 0.05% EDTA solution) for 10–15 min (37 °C, 5% CO_2_) and subsequent centrifugation (200 g, 5 min, room temperature). Cisplatin was added to the A2780cis cell line (at a concentration of 1 μM) every 2–3 passages to retain its resistance to this drug. Before every experiment, cell viability was assessed with by trypan blue exclusion (>95%). All cell lines were regularly tested with PlasmoTest—*Mycoplasma* detection kit (Invivogen, San Diego, CA, USA) and were proved to be *Mycoplasma*-free. For all experiments, cells were harvested in the exponential growth phase and at 90% of confluence.

All cell lines were suspended in growth medium (RPMI 1640, 2 mM L-glutamine, 1 mM sodium pyruvate, 10% FBS, as well as 100 U of penicillin and 10 mg of penicillin) and seeded on 96-well plates (MTT assay) or 24-well plates (phospho-kinase array, Immunobloting-ECL, real time PCR) at a concentration of 5 × 10^4^ cells/well or 1 × 10^6^ cells/well, respectively. Cells were cultured for 24 h to allow them to attach to the surface of the wells (37 °C, 5% CO_2_). For the phospho-kinase array, cell cultures were harvested at this point. For the remaining assays, medium was replaced and cisplatin at concentrations of 1, 2, 5, 10, 20, 40, 60, 80, 100, 200 µM, or PD98059 at concentrations of 25, 50, 100, 200 µM or Triciribine at concentrations of 10, 25, 50, 100 µM, or S3I-201 at concentrations of 25, 50, 100, 200 µM were added to the cells for 48 h, or cells were left untreated. For some experiments, the cell lines were pretreated with PD98059 or Triciribine or S3I-201 for 1 h before adding the cisplatin. Cell lines were then used in the MTT assay, Immunobloting-ECL or real-time PCR.

### 4.3. Human Phospho-Kinase Array—Proteome Profiling

Cells were harvested and lysed according to the manufacturer’s protocol—proteome profiler R&D (USA). In short, cells were rinsed with PBS, centrifuged and solubilized in lysis buffer at 1 × 10^7^ cell/mL for 30 min at 2–8 °C and centrifuged at 14,000× *g* for 5 min. Supernatants were transferred to clean Eppendorf tubes and kept at −80 °C until further analysis. Prior to the experiment, the amount of protein in each sample was measured with a DC protein assay kit. The membranes included in the kit were blocked with an appropriate blocking buffer for 1 h at room temperature, and next, an equal amount of protein for each tested cell line was added to membranes and they were incubated overnight at 2–8 °C. Membranes were then washed three times with wash buffer and incubated with a detection antibody cocktail for 2 h at room temperature. Afterwards, membranes were washed three times with a wash buffer and a chemi reagent mix was added to each membrane for 1 min. Next, membranes were exposed to X-ray film and the obtained protein dots were analyzed with FluoroChem MultiImage FC Cabinet (Alpha Innotech, San Leonardo, CA, USA), and their densitometry was measured with Alpha Ease FC software 3.1.2. Data are presented as optical density intensity (ODI) of the area under each dot’s peak.

### 4.4. MTT Assay

After treating cells with PD98059 or Triciribine, or S3I-201, or cisplatin, or a combination of PD98059 or Triciribine, or S3I-201 and cisplatin, supernatants were removed and 100 µl of MTT solution (2 mg/mL) was added to each well. Cells were incubated for 2–3 h at 37 °C with a 5% CO_2_, MTT was then gently removed, and 200 µl of 2-propanol was added. Plates were kept on rock platform for 15 min and absorbance was measured with the Multiskan RC plate reader (Labsystem, Helsinki, Finland) with a dual wavelength of 595 and 630 nm using Genesis Lite software. Cell line viability is presented as the percentage of cytotoxicity, calculated according to the formula

$$\mathrm{Cytotoxicity}\;\%=\left(1-(\frac{\mathrm{optical}\;\mathrm{density}\;\mathrm{of}\;\mathrm{sample}}{\mathrm{optical}\;\mathrm{density}\;\mathrm{of}\;\mathrm{control}})\right)\;\times \;100$$

EC_50_ values for each cell line were calculated by plotting a dose–response curve with the use of a GraphPad Prism 8 for Windows. Cisplatin in concentration equal to EC_50_ value was used to determine the impact of PD98059 or Triciribine or S3I-201 treatment on cells’ response to cisplatin.

### 4.5. Immunobloting ECL

Cell lines treated with PD98059 or Triciribine, or S3I-201, or left untreated, were gently harvested from 24-well plates, centrifuged (14,000× *g*, 2 min) and lysed with RIPA lysis buffer with the addition of 1 mM PMSF and 1% of Halt protease and phosphatase inhibition cocktail for 30 min on ice. The samples were centrifuged at 14,000× *g* for 5 min; next, supernatants were transferred to clean Eppendorf tubes and kept in −80 °C until further analysis. The amount of protein was measured with a DC protein assay kit for each sample. The lysates with equal level of proteins were put on a 10% SDS-PAGE mini-protean TGX gel for electrophoresis. Next, proteins were transferred to PVDF membranes using a Trans-Blot Turbo Transfer System (BioRad, Hercules, CA, USA) at 2.5 A for 10 min at room temperature. Afterwards, PVDF membranes were blocked with SuperBlock blocking buffer at a rocking platform for 30 min at room temperature, and then they were blotted with mouse monoclonal anti-SNAIL1 (1:1000), rabbit monoclonal anti-SNAIL2 (1:1000) or mouse anti-β-actin (1:4000) antibody for 1 h at room temperature. Next, membranes were washed five times (2 × TBS buffer with Tween 20) and blotted with HRP-conjugated goat anti-rabbit IgG (1:4000) or HRP-conjugated goat anti-mouse IgG (1:4000) for 1 h at room temperature. Subsequently, membranes were again washed five times (2 × TBS buffer with Tween 20) and shortly incubated with ECL Western Blotting Substrate in order to detect the selected proteins. Proteins were visualized by exposing membranes to X-ray films and densitometric analysis of blots was performed with FluoroChem MultiImage FC Cabinet. The results are presented as the optical density intensity (ODI) of the area under each band’s peak.

### 4.6. Total RNA Isolation and Real-Time PCR Analysis

Total RNA was isolated from cells using TRIzol^®^Reagent (Life Technologies, Thermo Fisher Scientific, Foster City, CA, USA). Isolation was performed according to the manufacturer procedures. Next, the quality control of isolated RNA was conducted on the Nanodrop with ND 1000 Software (Thermo Fisher Scientific, Waltham, MA, USA). RNA (5 μg) was processed directly to cDNA synthesis using Maxima First Strand cDNA Synthesis Kit for RT-qPCR (Life Technologies), according to the manufacturer’s manual. The expression of human *β-actin*, *GAPDH*, *SNAI1* and *SNAI2* was quantified using TaqMan Gene Expression Assays by real-time PCR, using ABI 7900-HT detection system (Applied Biosystems, Thermo Fisher Scientific, Foster City, CA, USA), according to the manufacturer’s protocol. Briefly, equal amounts of cDNA were amplified in triplicate for all TaqMan Assays in MicroAmp 96-well plates (Applied Biosystems). Each sample was supplemented with 1 μL of 20 × TaqMan Assay, made up to 20 μL using TaqMan 2 × Universal PCR Master Mix (with AmpErase UNG) and processed in the following steps: 50 °C—2 min, 95 °C—10 min, (95 °C—15 sec, 60 °C—60 sec) for up to 50 cycles. Each assay had controls with no template cDNA. The signal was collected at the endpoint of every cycle. Relative quantitation of gene expression was calculated using the comparative CT (∆∆CT) method [66]. The obtained data were analyzed with ABI 7900-HT (RQ manager software v1.2) and DataAssist software v3.01 and presented either as the relative RQ value, which represents the fold change in gene expression normalized to the reference genes (*β-actin* and *GAPDH*), or absolute 2^−∆CT^ value, which represents the absolute value of mRNA level of each evaluated gene in a particular cell line.

### 4.7. Flow Cytometry

Cell lines treated with PD98059 or Triciribine, or S3I-201, or left untreated were gently harvested from 24-well plates, centrifuged (200 g, 5 min, room temperature) and suspended in PBS with 2 mM EDTA and 0.5% BSA (1 × 10^5^ cells/100 µL). Cells were stained with E-cadherin-PE or N-cadherin-Alexa Fluor 488 antibody or the appropriate isotype control for 30 min in the dark, at room temperature, according to the manufacturer’s protocol. Afterwards, cells were washed once with PBS/2 mM EDTA and cells were resuspended in PBS/2 mM EDTA. Forward light scatter (FSC) and sideward light scatter (SSC) parameters were used to identify each ovarian cancer cell line population and to gate out debris. All samples were analyzed using a FACS BD LSR II flow cytometer (Becton Dickinson, Franklin Lakes, NJ, USA) equipped with BD FACS Diva Software. The results were analyzed with FlowJo v10.7.1 software (FlowJo, Becton Dickinson, Franklin Lakes, NJ, USA) and are presented as the median fluorescence intensity (MFI), which reflects the surface expression of the target proteins.

### 4.8. Statistical Analysis

Statistical evaluation of obtained results (presented as the mean ± SD) has been performed using data from either four or five different experiments, depending on the chosen statistical test. RT-PCR data were calculated by *t*-test with the use of DataAssist software v3.01 (Thermo Fisher Scientific, Waltham, MA, USA). The rest of the results were firstly analyzed with Shapiro–Wilk test to assess the normality of distribution and, according to these results, the nonparametric Wilcoxon’s singed rank test or Mann–Whitney *U* test were applied with the use of Statistica v13.0 for Windows (StatSoft, Krakow, Poland). Statistical significance was defined as *p* ≤ 0.05.

## 5. Conclusions

Our study indicates that SNAIL 1, but not necessarily SNAIL 2, may be involved in the ovarian cancer chemoresistance. Furthermore, STAT3 proved to be the most essential and universal factor determining the expression of SNAIL 1 and SNAIL 2 in ovarian cancer cells, regardless of their resistance to cisplatin or place of origin. Inhibiting STAT3 activity led to the so-called “cadherin switch”, as well as enhanced sensitivity to cisplatin in all tested cell lines. This may affect cells’ ability to undergo EMT as well as enhance cytotoxicity of chemotherapeutic agents. Our data encourage further, more thorough, investigation of SNAIL 1 and SNAIL 2 implications in cancer cells’ invasive behavior and resistance to chemotherapeutics, as well as searching for the potential agents responsible for the regulation of these transcription factors.

## Data Availability

Not applicable.

## Supplementary Materials

The following are available online at https://www.mdpi.com/1422-0067/22/2/980/s1, Table S1: The optical density intensity (ODI) values of signaling proteins of Proteome Profiler Human Phospho-Kinase Array in A2780, A2780cis, SK-OV-3 and OVCAR-3 cell lines.
