# Supplementary material for: Impact of Selected Signaling Proteins on SNAIL 1 and SNAIL 2 Expression in Ovarian Cancer Cell Lines in Relation to Cells’ Cisplatin Resistance and EMT Markers Level
Source: Int J Mol Sci. 2021 Jan 19;22(2):980. doi: 10.3390/ijms22020980 (PMC7835952; doi:10.3390/ijms22020980)
Supplement: Supplementary file 1 [file ijms-22-00980-s001.pdf]

Protein	ODI			
	A2780	A2780cis	SK-OV-3	OVCAR-3
**Reference**	**11026770**	**11226244**	**11514125**	**11177018**
PBS	107115	157914	166697	169541
ERK1/2 T202/Y204, T185/Y187	7825882	9203358	9954672	6940185
JNK1/2/3 T183/Y185, T221/Y223	3908515	3906894	5260240	3985368
AKT1/2/3 S473	9766970	9287656	9861440	9565985
GSK-3a/b S29/S9	9106000	8424904	9347780	9989698
β-catenin	1334500	1137264	4295520	8213919
p53 S392	3288365	2361947	1789015	10570496
CREB S133	8412060	9845667	10384855	10197990
c-Jun S63	1520545	2076814	4414055	7847964
STAT3 S727	2848940	3352733	7950850	5074474
WNK1 T60	7985805	6247599	7800040	7949527
PRAS40 T246	10073905	8750887	9972340	8634964
HSP60	6797718	6791721	8171065	7439511
p53 S46	3150205	2728780	2284350	2511959
p38a T180/Y182	1705805	1635634	2790675	1823479
EGF R Y1086	1014220	999191	1348630	1717234
AMPKa1 T183	2257660	1304749	1684610	1493726
HSP27 S78/S82	1646076	1968153	1797650	2254755
AMPKa2 T172	1795295	1625013	2051990	1929724
p53 S15	1874580	1754099	1874580	1927650
Src Y419	2011170	1785145	3428095	4483539
Lyn Y397	1374535	1232036	1566860	1759732
Lck Y394	1257570	1140532	1389450	1318225
STAT2 Y689	1958575	1950996	2354215	2131196
STAT5a Y694	1321155	1241840	1537815	1564556
RSK1/2/3 S380/S386/S377	1799596	1667497	2393465	2347045
eNOS S1177	1854955	1678118	2210560	2294892
Fyn Y420	1318015	1174029	1460100	1428405
Fgr Y412	1242655	1150336	1204190	1141150
STAT6 Y641	1667340	1713249	2081035	1691263
STAT5b Y699	1520545	1286775	1466380	1542520
p27 T198	1323510	1477136	1686180	2343985
PLC-y1 Y783	1870655	2129919	2165030	2747417
Hck Y411	1701095	1644621	2006460	1971435
Chk-2 T68	1807070	1774524	2313395	4166123
PDGF Rb Y521	1019715	1104584	1109205	1296976
STAT5 a/b Y694/Y699	2162675	2259005	2386400	2279152
PYK2 Y402	1714440	1683020	1673620	2263412
MSK1/2 S376/S360	1542525	1967336	3482224	2215405
TOR S2448	2658795	2434660	3120375	2711215
p70 S6 Kinase T389	2266295	2174854	2354215	3367573
p70 S6 Kinase T421/S424	3939130	3848070	2535550	4226977
Yes Y426	1798435	1643804	3775065	3605524
FAK Y397	3030885	2735316	2214485	2106012
